# Adiponectin expressing skeletal stem/progenitor cells in the bone and bone marrow homeostasis

**DOI:** 10.1016/j.coemr.2024.100524

**Published:** 2024-06

**Authors:** Biagio Palmisano, Alessandro Corsi, Mara Riminucci

**Affiliations:** 1 Department of Radiology, Oncology and Pathology, Sapienza University of Rome, 00161 Rome, Italy; 2 Department of Molecular Medicine, Sapienza University of Rome, 00161 Rome, Italy

**Keywords:** Bone marrow stroma, MSCs, Bone marrow adipocytes, Osteogenesis, Bone remodeling, RANKL, Fibrous dysplasia

## Abstract

The bone marrow stroma supports hematopoiesis while replenishing osteoblasts and adipocytes. These functions rely on different stromal cell populations identified by lineage tracing, phenotypic markers and single-cell transcriptomic analysis. A marrow stromal cell subset expressing *Adiponectin* (Adipoq^+^) has been reported by different studies in mice. However, while there is a general agreement on the kinetics of Adipoq^+^ stromal cells during mouse growth, their ability to generate bone marrow adipocytes and to support the bone vasculature, contrasting results have been reported on their osteogenic activity.

In this work, we review available data on Adipoq^+^ stromal cells, with special focus on the experimental evidence demonstrating their osteoprogenitor function and the potential reasons for the divergence observed among different studies. Furthermore, we discuss the potential overlap of Adipoq^+^ cells with other cell populations in the context of the widely recognized adipogenic bias that characterizes many marrow stromal stem cell populations.

## Bone marrow skeletal stem/progenitor cells

The postnatal bone marrow stroma is a reservoir of stem cells capable of replenishing osteoblasts and adipocytes while supporting hematopoiesis. The first evidence of their existence was provided a long time ago in ex-vivo transplantation studies [1]. However, their *in situ* identity remained elusive for a long time until studies in humans and mice (reviewed in Donsante et al. [2]) identified the perivascular fraction of the bone marrow stroma as the repository of its staminal and regulatory functions. This confirmed the long-surmised role of bone marrow pericytes in skeletal tissue regeneration and bone marrow homeostasis [3]. Regrettably, it has also raised the hypothesis that their properties could be shared by pericytes residing in different postnatal mesodermal tissues, leading to the concept of “mesenchymal stem cells” (MSCs) [3]. However, while on the one side attempts were made to show the existence of a single type of MSC distributed across the body later disproved by *in vivo* studies [4], on the other side evidence was gained regarding existence of multiple types of skeletal stem/progenitor cells (SSPCs) distributed across the skeleton. Indeed, over the last years, combinations of different techniques, such as cell sorting, *in vivo* transplantation assays, single cell RNA sequencing analysis (scRNA seq) and lineage tracing studies in mice, revealed the existence of different SSPC lineages within the bone marrow as well as the presence of SSPCs in other skeletal compartments including periosteum and growth plate [2,5]. Furthermore, scRNA seq also suggested unexpected features in some bone marrow SSPC populations, such as the presence of a phenotypic bias in multipotent cells. In this complex scenario, *Adiponectin*-expressing (Adipoq^+^) marrow stromal cells were identified in mice through scRNA seq and *Adipoq-Cre* recombination as a cell population appearing in the postnatal life around blood vessels and expanding within the marrow stroma during skeletal growth [6]. First thought of as adipocyte-restricted progenitors with local regulatory functions [6,7], Adipoq^+^ stromal cells were then recognized as a multipotent population of SSPCs [8,9] generating both osteoblasts and bone marrow adipocytes (BMAds, [10]) and playing a role in the homeostasis of the entire postnatal bone/bone marrow organ.

## Adipoq^+^ marrow stromal cells

**Figure 1:**
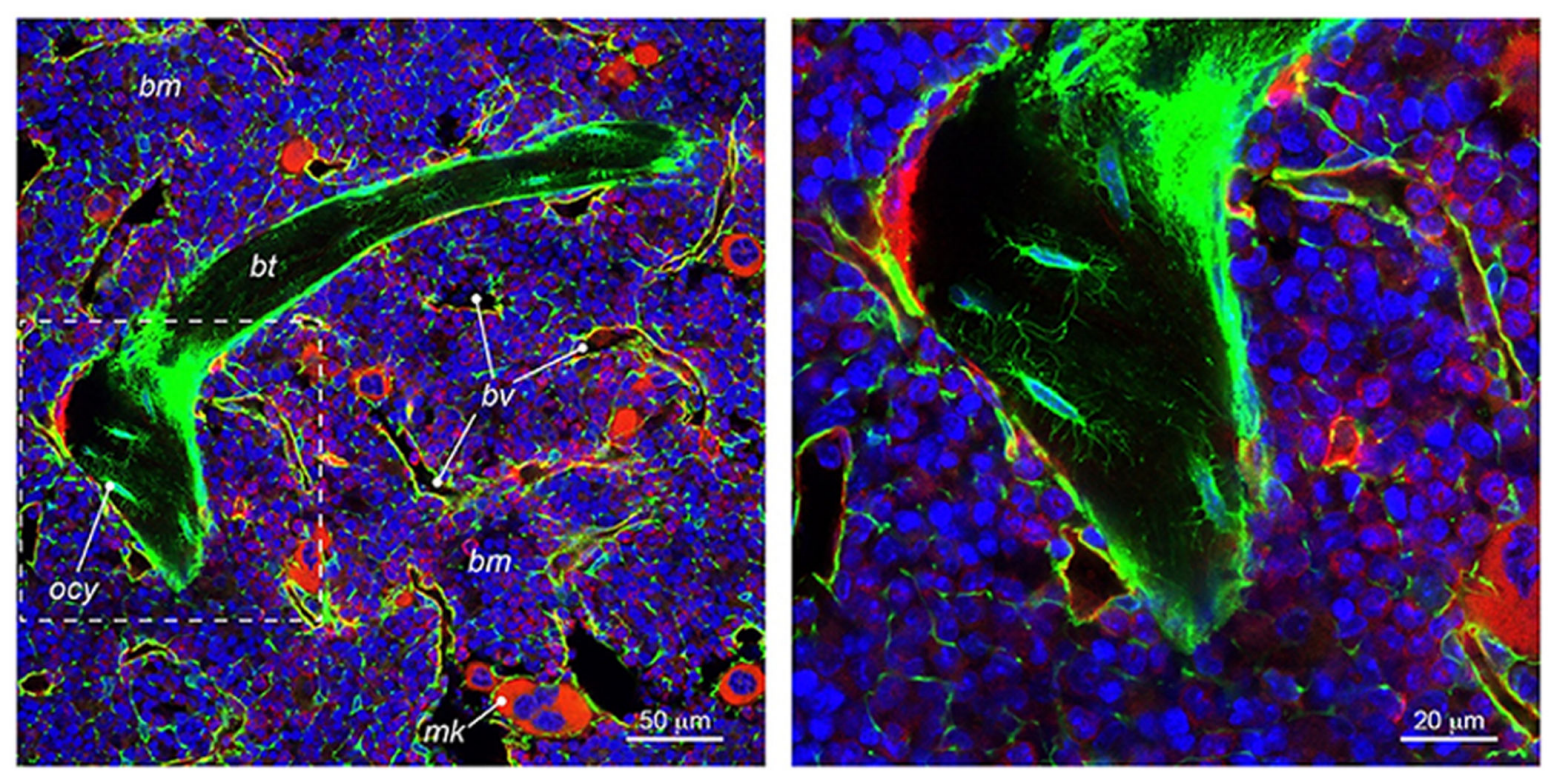
Confocal microscopy images of the bone marrow of a 12-month-old female *Adipoq-Cre;mTmG*^*het*^ (Tg (Adipoq-cre)1Evdr; Gt (ROSA)26Sortm4 (ACTB-tdTomato,-EGFP)Luo/J) mouse. In this mouse model, all *Adiponectin*-expressing cells and their lineage trajectories are marked by GFP and appear green, while all the other cells express TOMATO and appear red. Adipoq^+^ stromal cells are found around blood vessels and dispersed among hematopoietic cells. Moreover, GFP-marked osteocytes are found in bone trabeculae indicating that they arise from Adipoq^+^ SSPCs. Nuclei are stained with TO-PRO3. *bm* = bone marrow; *bt* = bone trabecula; *bv* = blood vessel; *ocy* = osteocyte; *mk* = megakaryocytes. (For interpretation of the references to color/colour in this figure legend, the reader is referred to the Web version of this article.)

The development of the Cre-lox recombination system greatly increased our understanding of the mouse bone marrow stroma by allowing the identification and tracing of cell populations marked by the expression of specific promoters. The existence of a bone marrow stromal cell subset undergoing *Adipoq-Cre* recombination was shown by multiple studies [6,8,11,12]. As common findings, these studies reported that Adipoq^+^ marrow stromal cells appear at early stages of postnatal life and progressively expand during mouse growth [6,8,12]. Overall, they form a 3D network that connects bone marrow and bone (Figure 1), being localized in the bone marrow cavity, around the vasculature and among hematopoietic cells, and, to a lesser extent, in the wall of blood vessels running through the cortical bone [6,8]. However, contrasting data were reported on their fate. Zhong et al. performed a single cell transcriptomic (scRNA seq) analysis showing that *Adipoq*^+^ stromal cells express adipocyte genes such as *Pparg, Cebpa, Apoe*, and *Lpl* but no other mature adipocyte markers (i.e., *Fabp4* or *Plin1* and lipid droplets), they are therefore positioned between “lineage committed progenitors” and differentiated adipocytes [6]. Moreover, through lineage tracing and transplantation experiments they concluded that Adipoq^+^ stromal cells represent a distinct population of progenitors generating adipocytes but not osteoblasts and called them marrow adipocyte lineage progenitors (MALPs) [6]. However, parallel and subsequent work performed by other groups using the same experimental approaches showed that *Adipoq-Cre* targeted stromal cells also have an osteogenic activity [8,9,12].

## Adipoq^+^ marrow stromal cells as osteoprogenitors

Discordant results on bone cell targeting in *Adipoq-Cre* lineage tracing experiments could be ascribed, in principle, to the use of different *Cre* recombination systems. However, most of the studies were performed on the constitutive *B6.FVB-Tg(Adipoq-cre)1Evdr/J* mouse model [13] indicating that the different patterns of cell labeling depended on other experimental factors. In particular, *Adipoq*-*Cre* labeled osteoblasts and osteocytes were not detected in studies in which only young mice were analyzed consistent with the evidence that the number of *Adipoq-Cre* targeted bone cells increases during mouse growth [8,12]. Other studies [6,9] used the inducible *B6*.129-Tg(Adipoq-cre/Esr1*)1Evdr/J mouse model [14]. They also reported opposite conclusions on osteoblast labeling possibly caused by using different tamoxifen induction doses. Additional details on these and other existing *Adipoq-Cre* mouse models are reported in Table 1.

As for scRNA seq, it is known that this approach may lead to misleading conclusions for technical reasons, e.g. false negative for low abundance RNAs. Most important, it poses intrinsic challenges in data interpretation since the analysis provides a snapshot of gene expression at a given time point, i.e. it detects a cell state, and therefore trajectory inferences do not necessarily reflect the developmental and functional context occurring *in vivo* [15]. Finally, the different results on the osteogenic activity of Adipoq^+^ stromal cells in some transplantation studies [6,8,16] likely resulted from the application of different osteo-conductive conditions.

*Adipoq-Cre* targeted osteoblasts and osteocytes are very low in young mice and progressively increase over time in parallel with the expansion of the Adipoq^+^ stromal cell network [8,12] (Figure 1). They distribute according to defined spatial pattern, being detected in appendicular and axial skeletal segments, but not in calvaria bones or in tail vertebrae, despite the presence of Adipoq^+^ stromal cells at these skeletal sites [8]. Furthermore, *Adipoq-Cre* targeted osteoblasts and osteocytes are found in the trabecular bone [8,12] and sparsely in cortical bone but not in the periosteum [8,9]. Thus, the Adipoq^+^ SSPC population is comprised of osteoprogenitors that do not contribute to embryonic/fetal skeletal development, but, according to their postnatal kinetics, participate in the growth and remodeling of many postnatal bones. Interestingly, the evidence of an osteogenic activity provides an alternative reading key for the absence of Adipoq^+^ stromal cells in fat depots other than the bone marrow [17]. The lack of an *Adipoq*-expressing precursor in peripheral fat was initially explained based on the incapacity of *Adipoq-Cre* cells to generate osteoblasts which, in the frame of the MSC concept, excluded them from the ubiquitous, non-tissue specific, network of multipotent perivascular progenitor cells. However, the intrinsic capacity of Adipoq^+^ marrow stromal cells to generate osteoblasts provides an alternative, reasonable explanation for their absence in fat depots outside bone, i.e., where osteoblast formation is not a canonical differentiation pathway and never occurs in physiological conditions.

## Adipoq^+^ SSPCs as bone marrow adipocyte progenitors

Adipoq^+^ SSPCs also act as progenitors of BMAds. Zhou et al. first reported that in *Adipoq-cre/ER;R26-tdTomato* mice receiving tamoxifen at 6 weeks of age, the number of TOMATO^+^/LEPR^+^ marrow stromal cells increased over time in parallel with the number of labeled BMAds [11]. In agreement, a following study by Zhong et al. identified *Adipoq*-expressing MALPs as progenitors of BMAds [6]. Due to the multiple functions of the bone marrow adipose tissue (BMAT), tight regulation of their adipogenic activity is critical to bone marrow homeostasis. Available data suggest that extrinsic microenvironmental factors as well as intrinsic molecular perturbations affect differentiation of Adipoq^+^ SSPCs. For example, in irradiated mice, loss of hematopoiesis favors the adipogenic pathway [11]. In contrast, in *Adipoq-Cre;Gsα*^*R201C*^ mice, the expression of a gain-of-function mutation of Gsα with excess cAMP production stimulates osteogenic differentiation at the expenses of the adipogenesis [8]. However, it must be noted that recent studies suggest that Adipoq^+^ SSPCs are not the only progenitors of BMAds. Indeed, Zhang et al. showed a secondary adipogenic pathway can be activated in the bone marrow but not in other fat depots by depletion of Adipoq^+^ cells [18]. As major features, BMAds derived by *Adipoq*-negative progenitors are specialized in lipid storage, have a reduced expression of adipokines and localize in areas typically occupied by red marrow in mice, such as the mid-diaphyseal region of tibiae [18].

## Potential overlap of Adipoq^+^ SSPCs with other marrow SSPC populations

The ability of Adipoq^+^ SSPCs to generate both adipocytes and osteoblasts, poses important questions on their potential overlap with other SSPCs and, consequently, on the effective lineage commitment of MALPs. Many studies reported *Adipoq* expression in SSPCs identified by phenotypic markers and/or lineage tracing experiments. For example, Zhou et al. observed *Adipoq-CreERT* recombination in 5% of *Lepr-Cre* targeted stromal cells [11] whereas Jeffery et al. reported recombination of *Adipoq-Cre and Adipoq-CreERT* in all *Lepr-Cre* cells residing in the femoral marrow, but not in those located within the periosteum [9]. Mukohira et al. observed that *Adipoq-Cre* targeted the majority of PDGFRβ^+^VCAM-1^+^ stromal cells, which were also targeted by *Lepr-Cre* [12] and almost all CXCL12 abundant reticular (CAR) cells. In a transcriptomic analysis of all non-hematopoietic bone marrow cells, Baryawno et al. identified a *Lepr*/*Cxcl12*^+^ “MSC” cluster expressing *Adipoq* [19]. Finally, Baccin et al. detected the expression of *Adipoq*, although at different levels, in two stromal cell populations both with high transcriptomic similarity to CAR cells but with a different adipogenic (Adipo-CAR) or osteogenic (Osteo-CAR) profile [20].

Altogether these data suggest that *Adipoq* expression may reflect a general tendency of bone marrow SSPCs towards an adipocyte priming rather than an adipocyte-restricted commitment. In this context, it remains to be assessed whether MALPs, defined as Adipoq^+^ bone marrow stromal cells, effectively represent a sub-population of committed adipocyte progenitors (as, presumably, Adipo-CAR) or if they coincide with the entire Adipoq^+^ cell population, being therefore able to differentiate into both adipocytes and osteoblasts.

## Adipoq^+^ marrow stromal cells as regulators of bone remodeling

In addition to their osteogenic activity, Adipoq^+^ stromal cells exert an indirect control on bone mass by acting on both branches of bone remodeling. Increased trabecular bone mass was observed in mice with ablation of either osteoclastogenic factors, such as RANKL and M-CSF [7,21,22] or osteogenic inhibitors, such as *Sclerostin*, specifically in *Adipoq-Cre* targeted cells [23]. However, since these cells give rise to adipocytes and osteoblasts, it remains to be clarified whether the observed bone mass changes must be ascribed to gene knock-down in Adipoq^+^ SSPCs or in their progeny. For example, the increase in bone mass in *Adipoq-Cre;Sost*^*f/f*^ transgenic model could be due to the lack of Sclerostin production by osteocytes, which are a major source of this molecule. The detection of the bone phenotype in mice older than 3 months of age, when *Adipoq-Cre* targeted osteocytes appear [8], and its absence in cranial bones, where Adipoq^+^ SSPCs do not differentiate into bone cells [8], seem to support this hypothesis.

Increased bone formation was also observed after ablation of *Adipoq-Cre* targeted cells via diphtheria toxin. Although Adiponectin is expressed by adipocytes in all fat depots, these changes were entirely dependent on the loss of Adipoq^+^ cells in the bone marrow, since transplantation of white peripheral adipose tissue rescued the metabolic syndrome caused by lack of adipocytes but not the bone phenotype [6,18,24]. However, ablation of Adipoq^+^ cells by using common *Adipoq-Cre* mouse models does not allow to distinguish the effect of the absence of Adipoq^+^ progenitor cells from those caused by the lack of mature BMAds. Of note, a novel transgenic mouse model with specific targeting of BMAds was developed [25] showing that ablation of constitutive BMAT by diphtheria toxin caused a local increase in bone formation. This revealed an important contribution of constitutive BMAds in bone mass regulation [26].

## Adipoq^+^ marrow stromal cells as regulator of marrow vascularity and cell composition

In the mammalian bone marrow, the intimate association of stromal progenitor cells with blood vessels is not merely an anatomical coincidence. It is rather a strategic position that allows them to perform specific regulatory functions. For example, differentiation into adipocytes at perivascular sites is itself a kind of regulatory function since adipocyte pressure may reduce the lumen of small blood vessels and sinusoids helping to shift blood flow from one area to another. Most important, the recruitment of SSPCs to the blood vessel wall often implies a role in bone marrow homeostasis that is independent of their differentiation. In the human bone marrow indeed, perivascular SSPCs support the development and maintenance of a normal marrow architecture and map the sites at which hematopoiesis appears by diffusible factors and cell–cell contacts [27]. Similarly, peri-vascular Adipoq^+^ stromal cells in mouse bone marrow are required to maintain a normal structural organization of the vascularity and of the marrow cavity at large. This is demonstrated by the dramatic consequence of their ablation, which is associated with reduced vascular density, abnormal morphology/dilation of individual blood vessels and enhanced intramedullary bone formation [6]. Adipoq^+^ marrow stromal cells are also involved in the development of specific hematopoietic cell subsets. In particular, they are a major source of IL-7 which is required for lymphocyte formation and maintenance [12]. Furthermore, Adipoq^+^ stromal cells regulate osteoclast formation and function through both cellecell contacts [7] and soluble mediators, especially RANKL [7,8,16] and M-CSF [21,22], but also *Il34, Ccl2, Vcam1* and *C3* whose transcripts are enriched in Adipoq^+^ stromal cells [7]. Interestingly, in spite of their wide distribution, the osteoclastogenic activity of Adipoq^+^ stromal cells is spatially restricted and does not affect osteoclast formation at sites such as the osteochondral junction and the cortical bone [7]. However, it must be noted that *Adipoq-Cre;Gsα*^*R201C*^ mice develop perivascular lytic lesions in the bony cortex [8]. This suggests that osteoclast stimulation is an inherent ability of all Adipoq^+^ stromal cells but there are some sites at which it serves specific physiological transient functions (e.g., shaping of cortical vascular canals) and cannot be appreciated in steady state conditions.

## Bone regenerative potential of Adipoq^+^ SSPCs

The skeletal regeneration activity of bone marrow and periosteal SSPCs is commonly assessed on long bone following cortex disruption by either drill hole or fracture. Using these models it was shown that bone marrow Lepr^+^/Adipoq^+^ SSPCs, contribute primarily to bone healing after drill injuries [9], a condition in which cortical bone and the endosteal surface are removed and regeneration occurs through intra-membranous ossification.

Previous work reported that bone repair in this model was carried out by *Cxcl12*-targeted SSPCs, which do not generate cortical bone osteocytes in physiological conditions [28]. This is consistent with the evidence that Adipoq^+^ stromal cells represent a fraction of Cxcl12^+^ cells [20,28]. Other SSPC populations are also involved in cortical regeneration after drill hole injury such as *Osx-Cre* targeted cells [28].

In contrast Lepr^+^/Adipoq^+^ cells do not provide a significant regenerative contribution in the bicortical fracture model [9]. This type of injury is repaired through endochondral ossification by periosteal SSPCs, such as *Gli1-Cre* labeled cells [9]. This seems to be in contrast with previous studies showing that Lepr^+^ SSPCs differentiate, after the fracture, into chondrocyte in the soft callus and into osteoblasts and osteocytes in newly-formed bone [29]. However, it must be noted that *Lepr-Cre* recombined not only in bone marrow SSPCs but also a in small subset of periosteal SSPCs [9,30,31]. A contribution to bone healing in the fracture model is also provided by *Cxcl12-Cre* targeted stromal cells that differentiate into Sox9^+^ chondrocytes [28].

## Adipoq^+^ marrow stromal cells in skeletal diseases

Conditional ablation of specific molecules revealed the function of Adipoq^+^ SSPCs in preserving bone integrity [7,16,21,22] whereas other studies showed the importance of this cell population in mediating bone and bone marrow repair after injury [32]. However, whether and how Adipoq^+^ SSPCs may play a direct role in the pathogenesis of bone and bone marrow diseases is still largely unknown. A first evidence was provided by *Adipoq-Cre;Gsα*^*R201C*^ mice. Consistent with the presence of Adipoq^+^ perivascular cells around intraosseous blood vessels these mice developed a skeletal phenotype reminiscent of the human disease associated with gain-of-function mutations (R201C, R201H) of Gsα, named Fibrous dysplasia (FD) of bone (OMIM #174800) [33]. Specifically, they showed lytic lesions in both cortical bone, with increased porosity, and trabecular bone, with tunneling resorption [8]. Interestingly, these lesions were predominantly observed in skeletal segments and compartments in which Adipoq^+^ stromal cells do not have any osteogenic activity (e.g., tail vertebrae), thus confirming that, at sites in which they behave as osteoprogenitor cells (e.g., femur metaphysis), bone formation prevails on the stimulation of bone resorption. Although further studies are required to better understand the mechanism of lesion development in *Adipoq-Cre;Gsα*^*R201C*^ mice, these data strongly suggest that Adipoq^+^ marrow stromal cells could be implicated in the pathogenesis of FD and other skeletal diseases with abnormally increased bone resorption such as, for example, hyperparathyroidism.

## Conclusions and future perspective

**Figure 2:**
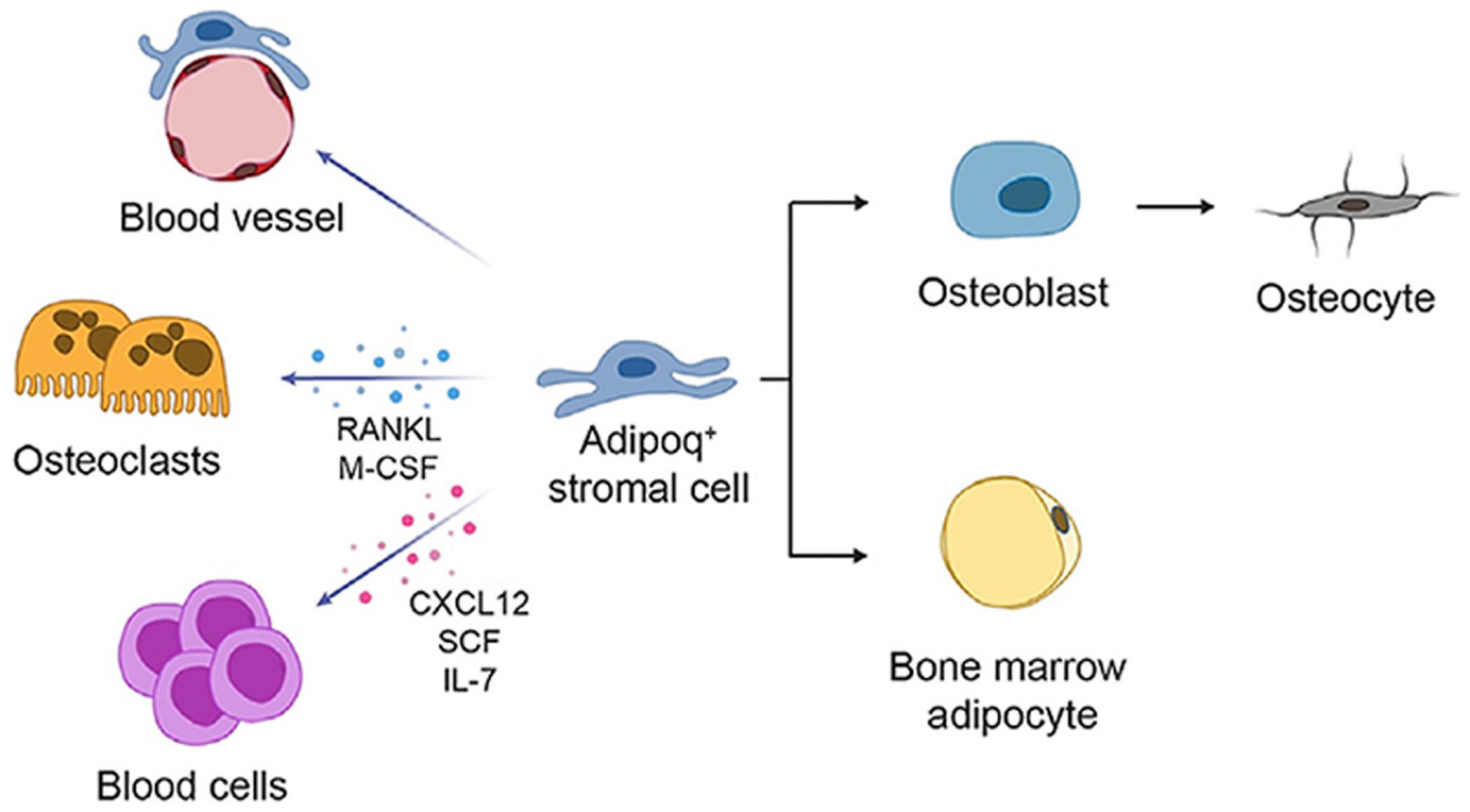
Schematic representation of Adipoq^+^ stromal cell functions and differentiation paths. This cell population regulates the blood vessels network in the bone marrow, stimulates osteoclast formation and differentiation through RANKL and M-CSF, and regulates hematopoiesis through CXCL12, SCF and IL-7. In addition, Adipoq^+^ SSPCs show a bi-lineage differentiation potential, generating both osteoblasts and BMAds.

In the postnatal skeleton, several local and systemic inputs continuously affect bone remodeling, marrow vascularization and hematopoiesis. Being involved in all these functions, Adipoq^+^ marrow stromal cells may help to coordinate different adaptive responses and to maintain bone and bone marrow homeostasis (Figure 2). However, many questions still need to be addressed on this cell population. Further studies are required to clarify their origin, whether from other stromal cell subsets or extra-skeletal cell populations, their relationship with other types of bone marrow SSPCs and their regulation. Furthermore, attention should be paid in the use of *Adipoq-Cre* systems, to better distinguish the functions of Adipoq^+^ SSPCs from those of their differentiated progeny, BMAds and bone cells. Finally, although a human scRNA seq dataset showed a stromal cell cluster with high levels of expression of *ADIPOQ* and *CSF1* [34], further studies are required to assess whether the human bone marrow effectively contains an Adipoq^+^ marrow stromal cell population equivalent in space, time and functions to that detected in mice.

## Figures and Tables

**Table 1:** *Adipoq* transgenic mice present in The Jackson Laboratory repository and studies that have used *Adipoq-Cre* mouse models to study bone and bone marrow cells.

Reference	Technique(s)	Study design	Adipoq-Cre Strain	Reported recombination in osteoblast/cytes
Mukohira et al., 2019 [12]	Lineage tracing; Genetic manipulation	*Adipoq-Cre;R26-tdTomato* *Adipoq-Cre;Il7 f/f*	A	Yes
Yu et al., 2021 [7]	Genetic manipulation	*Adipoq-Cre;Tnfsf11 f/f*	A	Yes
Zhang et al., 2021 [18]	Lineage tracing; Genetic manipulation	*Adipoq-Cre;R26*-lsl-*mTmG* *Adipoq-Cre;R26*-lsl-*DTA*	A	No
Palmisano et al., 2022 [8]	Lineage tracing; Genetic manipulation	*Adipoq-Cre;R26*-lsl-*Gs*□ *R201C* *Adipoq-Cre;R26*-lsl-*mTmG*	A	Yes
Inoue et al., 2023 [21]	Genetic manipulation	*Adipoq-Cre;Csf1 f/f*	A	Not specified
Zhong et al., 2023 [22]	Genetic manipulation	*Adipoq-Cre;Csf1 f/f*	A	Not specified
Zhong et al., 2020 [6]	Lineage tracing	*Adipoq-Cre;R26-tdTomato* *Adipoq-Cre;R26-tdTomato;R26*-lsl-*DTR*	A, C	No
Jeffery et al., 2022 [9]	Lineage tracing: drill injury	*Adipoq-Cre;R26-tdTomato* *Adipoq-CreERT;R26-tdTomato*	A, C	Yes
Zhong et al., 2022 [32]	Lineage tracing; radiation injury model	*Adipoq-Cre;R26-tdTomato* *Adipoq-CreERT;R26-tdTomato* *Adipoq-Cre;R26-tdTomato;R26*-lsl-*DTR*	A, C	Not specified
Zhang et al., 2019 [35]	Genetic manipulation	*Adipoq-Cre;Kitl f/f*	B	Not specified
Zou et al., 2020 [24]	Genetic manipulation	*Adipoq-Cre;R26*-lsl-*DTA*	B, E	Not specified
Li et al., 2022 [25]	Lineage tracing; Genetic manipulation	*Osterix-FLPo;FAC;R26*-lsl-*mTmG*	D	No
Zhou et al., 2017 [11]	Lineage tracing; Genetic manipulation; radiation injury model	*Adipoq-CreERT;R26-tdTomato;* *Adipoq-CreERT;Scf f/f*	E	Yes
Hu et al., 2021 [16]	Genetic manipulation	*Adipoq-Cre;R26-tdTomato* *Adipoq-Cre;Tnfsf11 f/f*	Not specified	No
Gao et al., 2023 [23]	Genetic manipulation	*Adipoq-Cre;Sost f/f*	Not specified	Not specified
Assigned letter	*Catalog at The Jackson Laboratory*		*Strain*	
A	028020		*B6.FVB-Tg(Adipoq-cre)1Evdr/J* [13]	
B	010803		*B6;FVB-Tg(Adipoq-cre)1Evdr/J* [13]	
C	024671		B6.129-Tg(Adipoq-cre/Esr1 *)1Evdr/J [14]	
D	037702		*B6.Cg-Adipoqem1(cre)Oam/J* [25]	
E	025124		C57BL/6-Tg*(Adipoq-icre/ERT2)1Soff/J* [36]	
F	033448		*C5*7BL*/*6-Tg*(Adipoq-rtTA)2Zvw/J* [37]	

The *B6.FVB-Tg(Adipoq-cre)1Evdr/J* model (A) has been previously reported to have highly efficient *Cre* recombinase activity in peripheral adipocytes and no ectopic expression [13]. The *B6;FVB-Tg(Adipoq-cre)1Evdr/J* model (B) is the non-congenic strain version of A. The B6.129-Tg(Adipoq-cre/Esr1*)1Evdr/J model (C), expresses a tamoxifen-inducible Cre recombinase under the *Adipoq* promoter [14]. This mouse model has been reported to have lower efficiency in targeting adipocytes in peripheral depots [14], and stromal cells in the bone marrow than A [9]. The mouse model *B6.Cg-Adipoqem1(cre)Oam/J* (D), has been recently generated with the aim to specifically target bone marrow adipocytes [25]. However, a small portion of bone marrow stromal cells was reported to be targeted [25]. The *C57*BL/6-Tg*(Adipoq-icre/ERT2)1Soff/J* mouse (E) has been reported to have efficient recombination in white adipose tissues [36] and it was used for bone marrow adipose tissue ablation experiments but it resulted much less effective than B [24]. Moreover, bone marrow stromal cells and rare osteoblasts has been found to be targeted by this *Adipoq-Cre* system [11]. The *C57*BL/6-Tg*(Adipoq-rtTA)2Zvw/J* mouse (F) has been used to target peripheral adipose tissues and no data are available on targeting of bone and bone marrow stromal cells.

## Data Availability

No data was used for the research described in the article.
